# Attention to the body depends on eye-in-orbit position

**DOI:** 10.3389/fpsyg.2014.00683

**Published:** 2014-07-08

**Authors:** Elena Gherri, Bettina Forster

**Affiliations:** ^1^Department of Psychology, University of Edinburgh, EdinburghUK; ^2^Department of Psychology, City University London, LondonUK

**Keywords:** endogenous tactile attention, somatosensory processing, somatotopic and external space, eye position, event-related potentials

## Abstract

Attentional selectivity in touch is modulated by the position of the body in external space. For instance, during endogenous attention tasks in which tactile stimuli are presented to the hands, the effect of attention is reduced when the hands are placed far apart than when they are close together and when the hands are crossed as compared to when they are placed in their anatomical position. This suggests that both somatotopic and external spatial reference frames coding the hands’ locations contribute to the spatial selection of the relevant hand. Here we investigate whether tactile selection of hands is also modulated by the position of other body parts, not directly involved in tactile perception, such as eye-in-orbit (gaze direction). We asked participants to perform the same sustained tactile attention task while gazing laterally toward an eccentric fixation point (Eccentric gaze) or toward a central fixation point (Central gaze). Event-related potentials recorded in response to tactile non-target stimuli presented to the attended or unattended hand were compared as a function of gaze direction (Eccentric vs. Central conditions). Results revealed that attentional modulations were reduced in the Eccentric gaze condition as compared to the Central gaze condition in the time range of the Nd component (200–260 ms post-stimulus), demonstrating for the first time that the attentional selection of one of the hands is affected by the position of the eye in the orbit. Directing the eyes toward an eccentric position might be sufficient to create a misalignment between external and somatotopic frames of references reducing tactile attention. This suggests that the eye-in-orbit position contributes to the spatial selection of the task relevant body part.

## INTRODUCTION

The term spatial attention refers to our ability to select stimuli from our perceptual environment that are presented at relevant locations. In the tactile modality, spatial attention allows us to prioritize the processing of stimuli that are presented at relevant body locations. Behavioral studies demonstrated that spatial attention facilitates speed and accuracy of responses to tactile stimuli when presented to the attended hand as compared to the unattended one (Sathian and Burton, 1991; Spence et al., 2000). This performance improvement is typically accompanied by increased brain activity for stimuli presented at attended body locations, as demonstrated by electrophysiological and neuroimaging studies. Event-related potential (ERP) investigations demonstrated that the early stages of somatosensory processing are modulated by tactile–spatial attention (Desmedt and Robertson, 1977; Michie, 1984; Michie et al., 1987; García-Larrea et al., 1995; Eimer and Forster, 2003; Forster and Eimer, 2004). In these studies, participants were instructed to attend to their left or right hand while a tactile stimulus was delivered to the attended or the unattended hand. ERPs elicited by attended stimuli were characterized by an amplitude enhancement of the mid-latency somatosensory ERP components, P100 and N140, followed by a sustained attentional negativity (Desmedt and Robertson, 1977; Michie, 1984; Michie et al., 1987; García-Larrea et al., 1995; Eimer and Forster, 2003; Forster and Eimer, 2004; Jones and Forster, 2013). The time course of these attentional ERP modulations indicates that the effects of tactile attention begin already in modality-specific somatosensory cortical areas. This is further supported by neuroimaging studies which suggested the involvement of the secondary somatosensory cortex in higher order processing for somatosensory perception (c.f. Mima et al., 1998; Burton et al., 1999).

One intriguing aspect of tactile spatial attention concerns the spatial representations (or reference frames) upon which it operates. Cortical representations of tactile information within the somatosensory cortex are somatotopically organized (cf. Merzenich et al., 1978; Kaas, 1983). These somatotopic representations encode body locations relative to the position of their mechanoreceptors on the skin. Because the separation between mechanoreceptors of different body parts within the correspondent representation on the cortical surface of the brain remains constant even when the body is moving, somatotopic representations are independent from the position of the body in external space (body posture). While in principle tactile attention could operate exclusively within such somatotopic representations of the body, a number of studies have now demonstrated that the effects of tactile attention are modulated by body posture (Driver and Grossenbacher, 1996; Eimer et al., 2001, 2003, 2004; Soto-Faraco et al., 2004; Röder et al., 2008; Gillmeister et al., 2010; Heed and Röder, 2010; Eardley and van Velzen, 2011; Gherri and Forster, 2012a; Gillmeister and Forster, 2012). In these experiments, the somatotopic (anatomical) separation between the relevant receptors (the hands) was held constant, while their separation in external space was manipulated.

For instance, Driver and Grossenbacher (1996) instructed their participants to discriminate a vibration delivered to the attended hand while ignoring a simultaneous vibration to the unattended hand. These simultaneous vibrations could be identical (congruent condition) or different (incongruent condition). Because tactile attention cannot be focused completely on the task-relevant hand, performance was impaired when incongruent vibrations were presented to the other hand (interference effect). Crucially, this interference effect was modulated by the spatial separation between hands. Stronger interference effects were observed when the hands were close together as compared to the condition in which the hands were positioned far apart, suggesting that tactile attentional selectivity is more efficient when the hands are far apart (Driver and Grossenbacher, 1996; see also Soto-Faraco et al., 2004, for similar findings with a different task). In addition, analogous results were described in ERP studies investigating the impact of the near vs. far hands posture manipulation on the attentional modulations of tactile processing (Eimer et al., 2004; Gillmeister et al., 2010; Gillmeister and Forster, 2012). Here, participants were instructed to attend to their left or right hand in order to discriminate a forthcoming tactile stimulus. ERPs elicited by attended and unattended tactile stimuli when the hands were near and far apart, revealed stronger N140 attentional modulations when the hands were wide apart (Eimer et al., 2004; Gillmeister et al., 2010; Gillmeister and Forster, 2012). The observation of a hand distance effect on tactile attention supports the hypothesis that attention in touch is not exclusively mediated by somatotopic representations of the body but also by spatial representations that encode the limb position in external space.

Further evidence that tactile attention operates on multiple spatial representations, comes from ERP studies in which tactile attention was investigated under uncrossed and crossed hands conditions. In the uncrossed hands condition, tactile spatial attention was found to modulate the P100 and N140 mid-latency components, as well as the later Nd component, with increased amplitudes for ERP components elicited by tactile stimuli delivered to the attended hand. In contrast, in the crossed hands condition, the P100 and N140 modulations were absent, and the amplitude of the Nd was strongly reduced as compared to the uncrossed hands condition (Eimer et al., 2001, 2003; Röder et al., 2008; Heed and Röder, 2010; Eardley and van Velzen, 2011; Gherri and Forster, 2012a). The fact that attentional ERP modulations were delayed and attenuated with crossed as compared to uncrossed hands has been interpreted as indicating a conflict between the somatotopic representations and the body representations that encode body locations according to external space. When the hands are crossed, the left hand is placed on the right side of external space and vice versa. Attentional selectivity might be therefore disrupted by the conflict between the competing somatotopic and external spatial representations (Eimer et al., 2001, 2003).

Taken together the effects of hand separation and hand crossing on tactile attention demonstrate that multiple spatial representations of the body are active during tactile attention tasks. Some of these representations encode relevant body locations according to somatotopic space while others encode the locations of task-relevant body parts with respect to external space. These external or “postural” representations (Medina and Coslett, 2010) encode postural changes of the body, providing information relative to the current location of relevant body parts in external space. External representations are likely to be related to an abstract “visual” representation of space (c.f. Driver and Grossenbacher, 1996; Röder et al., 2004, 2008; Soto-Faraco et al., 2004), which receives contributions not only from visual but also from proprioceptive and vestibular inputs.

Little is known about the external representations that mediate *tactile spatial attention*. One of the outstanding questions concerns the reference point(s) according to which body locations are encoded within these external representations. Several body parts such as the trunk (or body midline), the head or the eyes might operate as reference points (egocentric representations). Alternatively, the distance between relevant body parts might be coded according to their relative position in external space or according to an external object, such as the fixation point (allocentric representations)^1^. However, all these reference points are typically confounded in standard attention tasks in which participants are asked to keep their eyes on a central fixation point which is aligned with their body midline, head, and trunk.

The present study represents a first attempt to investigate systematically the reference point(s) of the external representation(s) that mediate endogenous tactile spatial attention. More specifically, we investigated whether the eyes are used as a reference point to encode the position of other body parts in external space during a tactile attention task. To this aim, we manipulated the position of the eyes (eye-in-orbit position or gaze direction^2^) while leaving all others reference points candidates aligned with each other. In different blocks of trials, participants performed the same tactile attention task while gazing laterally to an eccentric fixation point (Eccentric gaze condition) or toward a central fixation (Central gaze condition). In both conditions, the head and trunk were aligned with the central fixation point and the body midline. A sustained attention task was used to manipulate the spatial allocation of tactile attention. At the beginning of each block, participants were verbally instructed to covertly direct their tactile attention to one hand (the task-relevant hand, which remained constant throughout the same block of trials) in order to respond to infrequent target (“gap”) stimuli at that hand while ignoring all target stimuli presented to the opposite hand. In addition, they were instructed to ignore tactile non-target stimuli (no gap stimuli) presented to either hand. Because target and non-target stimuli were difficult to discriminate, participants were strongly motivated to focus and maintain their tactile attention on the task-relevant hand throughout the task. ERPs in response to tactile non-target stimuli presented to the attended or unattended hand were compared as a function of gaze direction (Eccentric vs. Central gaze). If eye-in-orbit position is used as a reference point to encode the location of the task-relevant hand during tactile attention tasks, attentional modulations of somatosensory ERPs should be influenced by the manipulation of gaze direction. In other words, any impact of eye-in-orbit position on tactile spatial attention would demonstrate a role of the eyes as reference point for tactile attention.

## MATERIALS AND METHODS

### PARTICIPANTS

Eighteen paid volunteers (seven males) aged 21–35 (mean age of 26.5 years) participated in the experiment. Two were left handed and they all had normal or corrected-to-normal vision by self-report. All participants gave written informed consent. The study was performed in accordance with the ethical standards laid down in the 1964 Declaration of Helsinki and was approved by the Ethics committee, Department of Psychology, City University London.

### STIMULI AND APPARATUS

Participants sat in a dimly lit experimental chamber. Tactile stimuli were presented using a 12 V solenoids, driving a metal rod with a blunt conical tip to the top segment of the index fingers, making contact with the fingers whenever a current was passed trough the solenoid. Two tactile stimulators were used, each attached with adhesive medical tape to the left and right index finger, placed so that the metal rod made contact with the outer side of the top phalanx.

Tactile stimuli were either continuous (non-target stimuli), consisting of one rod contacting one finger for 200 ms, or contained a 6-ms gap in which this contact was interrupted after a duration of 97 ms (target stimuli). Throughout the experimental blocks, white noise (62 dB SPL) was continuously delivered from a loudspeaker centrally located in front of the participants, to mask any sounds made by tactile stimulators.

Participants were instructed to place their hands palm side down on a table with their left and right index finger positioned 20 cm from the left and the right of the body midline. A black cardboard panel (69 cm × 41 cm) was placed on the table and was used to prevent the visibility of the hands and lower parts of the arms. One marker (white circle, 0.2 cm diameter) located at the center of this panel at a viewing distance of approximately 58 cm was used as a central fixation point when participants were instructed to gaze to the center (Central gaze condition). Two additional markers (white circles, 0.2 cm diameter) that were located 20 cm to the right or left of the central marker were used as lateral fixation points when participants were instructed to gaze to the left or right side of space (Eccentric gaze condition). Participants head and body midline were aligned with each other and with the central fixation.

### PROCEDURE

Each trial started with a 200 ms tactile stimulus presentation (either target or non-target) followed by a 1000 ms interval used to collect vocal responses. Intertrial interval was varied randomly between 200 and 300 ms.

The experiment consisted of 18 blocks, with 64 trials per block. In each block, a non-target stimulus was presented with equal probability to the attended or to the unattended hand on 48 trials (24 to the attended hand and 24 to the unattended hand). A target stimulus was presented in the remaining 16 trials. Of these, twelve were trials in which a target stimulus was presented to the attended hand, while four were trials in which a target stimulus was presented to the unattended hand. Participants were instructed to respond vocally (by saying “yes”) whenever a target stimulus was presented to the attended hand, they had to ignore target stimuli to the unattended hand as well as all non-target stimuli. At the beginning of the session, a block of trials was run to familiarize participants with the task and the stimuli.

At the beginning of each block, participants were instructed about the task-relevant hand and the relevant gaze direction. They had to maintain their covert tactile attention and gaze on the instructed locations throughout the block of trials. On twelve successive blocks, participants executed the sustained attention task while gazing laterally (Eccentric gaze condition). In this condition, they gazed toward the left fixation point for six blocks and to the right fixation point for the remaining six blocks. On half of these blocks the left hand was task-relevant, while on the other half the right hand was task-relevant. The order of task-relevant hand (left vs. right) and gaze direction (left vs. right) in which these blocks were delivered was counterbalanced across participants. On the remaining six successive blocks, participants performed the same sustained attention task whilst maintaining their gaze on a central fixation point (Central gaze condition). The order of task-relevant hand (left vs. right) in which these blocks were delivered was counterbalanced across participants. Half of the participants started with the Eccentric Gaze condition, while the other half first performed the Central gaze condition.

Participants’ gaze direction was monitored via a video camera throughout the experiment.

### EEG RECORDING AND DATA ANALYSES

Electroencephalography (EEG) WAS recorded from 28 Ag–AgCl electrodes (Fp1, Fp2, F7, F8, F3, F4, Fz, Fc5, Fc6, Fc1, Fc2, Fcz, T7, T8, C3, C4, Cz, Cp5, Cp6, Cp1, Cp2, P7, P8, P3, P4, Pz, O1, O2) relative to a right earlobe reference. Horizontal EOG was recorded unipolarly from the outer canthi of both eyes. Electrode impedance was kept below 5 kΩ, and the impedance of the earlobe electrodes was equalized as much as possible. EEG was amplified, band-pass filtered at 0.01–100 Hz, digitized at 500 Hz, and filtered off-line with a lowpass filter of 30 Hz. EEG was digitally re-referenced to the average of the left and right earlobe and HEOG was averaged for the left and right eye.

Trials with eyeblinks (voltage at Fp1 and Fp2 exceeding ±60 μV), horizontal eye movements (voltage at HEOG exceeding ±30 μV), or other artifacts (voltage at any site exceeding ±60 μV) were excluded prior to data analysis, as were trials with response errors. The average rate of excluded trials was 6.5% across all participants.

Event-related potentials to non-target stimuli were averaged relative to a 100 ms pre-stimulus baseline for 300 ms after stimulus onset, separately for all combinations of attended hand (left vs. right), gaze direction (Eccentric gaze, collapsed across left and right fixation blocks, vs. Central gaze) and stimulus location (left vs. right). To obtain fine-grained time course information about the effects of gaze direction on the attentional modulations of somatosensory processing, ERP mean amplitudes were computed within successive 30 ms measurement windows starting from 20 to 260 ms after stimulus onset (20–50 ms; 50–80 ms; 80–110 ms; 110–140 ms; 140–170 ms; 170–200 ms; 200–230 ms; 230–260 ms). Analyses of somatosensory ERPs were conducted separately for lateral anterior (F7/8, F3/4, and FC5/6), lateral central (FC1/2, C3/4, and CP1/2) and lateral posterior (P7/8, P3/4, and CP5/6) sites, contralateral and ipsilateral to the stimulated hand, and for midline sites (Fcz, Cz, and Pz).

To investigate whether the manipulation of the eye-in-the-orbit position affects ERPs attentional modulations, somatosensory ERPs in response to tactile non-target stimuli delivered to the attended vs. unattended hand were compared, as obtained in the Eccentric gaze and Central gaze conditions. This analysis included the factors gaze direction (Eccentric vs. Central gaze), attention (stimuli presented to the attended vs. unattended hand), stimulus location (left vs. right), and electrode site (F7/8, F3/4, and FC5/6 for lateral anterior electrodes; FC1/2, C3/4, and CP1/2, for lateral central electrodes; P7/8, P3/4, and CP5/6 for lateral posterior electrodes and Fcz, Cz, and Pz for midline electrodes).

When appropriate, Greenhouse–Geisser adjustments to the degrees of freedom were performed.

The latency of vocal responses was measured with a voice key relative to the gap onset of the target stimuli (97 ms after stimulus onset), as target/non-target discriminations were only possible after this interval. For vocal responses to attended tactile targets, mean response times (RTs) for each participant was calculated for each condition (Eccentric vs. Central gaze).

## RESULTS

### BEHAVIORAL RESULTS

In this analysis, RTs to attended stimuli recorded in the Eccentric gaze condition were compared to those recorded in the Central gaze condition. A tendency emerged for vocal responses to be faster when participants gazed to the center than when they gazed laterally. This tendency marginally approached significance (461 ms and 484 ms, respectively, *F*(1,17) = 4, *p* = 0.06, $\eta_{p}^{2}$ = 0.19).

False alarms to non-targets occurred on 0.3% of all non-target trials. Participants responded to targets presented to the unattended hand on 0.15% of these trials, while they missed 2.6% of all tactile targets presented to the attended hand. None of these measures was affected by gaze direction.

### EEG RESULTS

**FIGURE 1:**
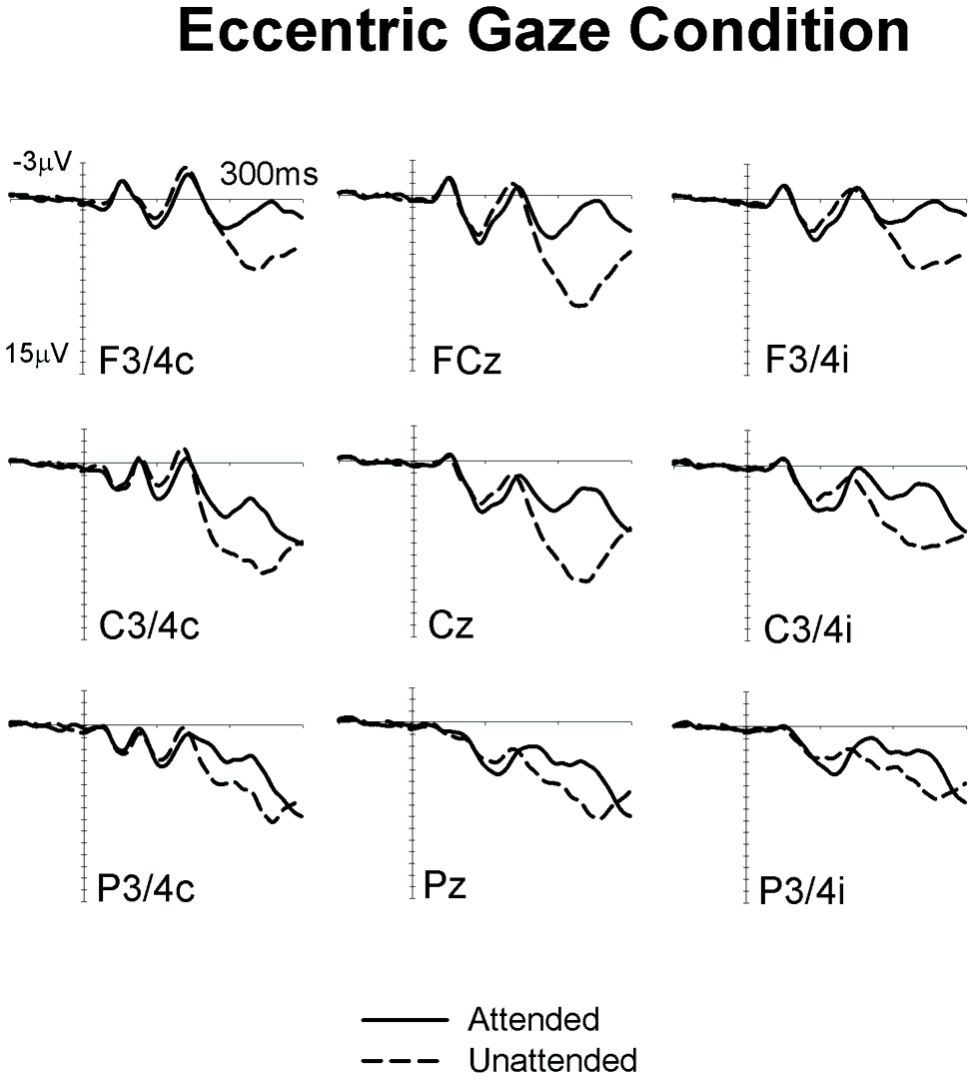
**Grand-averaged somatosensory ERPs elicited in the Eccentric gaze condition at midline electrodes, and at sites contralateral (C) and ipsilateral (I) to the stimulated hand, by tactile non-target stimuli presented to the attended hand (solid lines) and to the unattended hand (dashed lines) in the 300 ms following stimulus onset (relative to a 100 ms pre-stimulus baseline)**.

**FIGURE 2:**
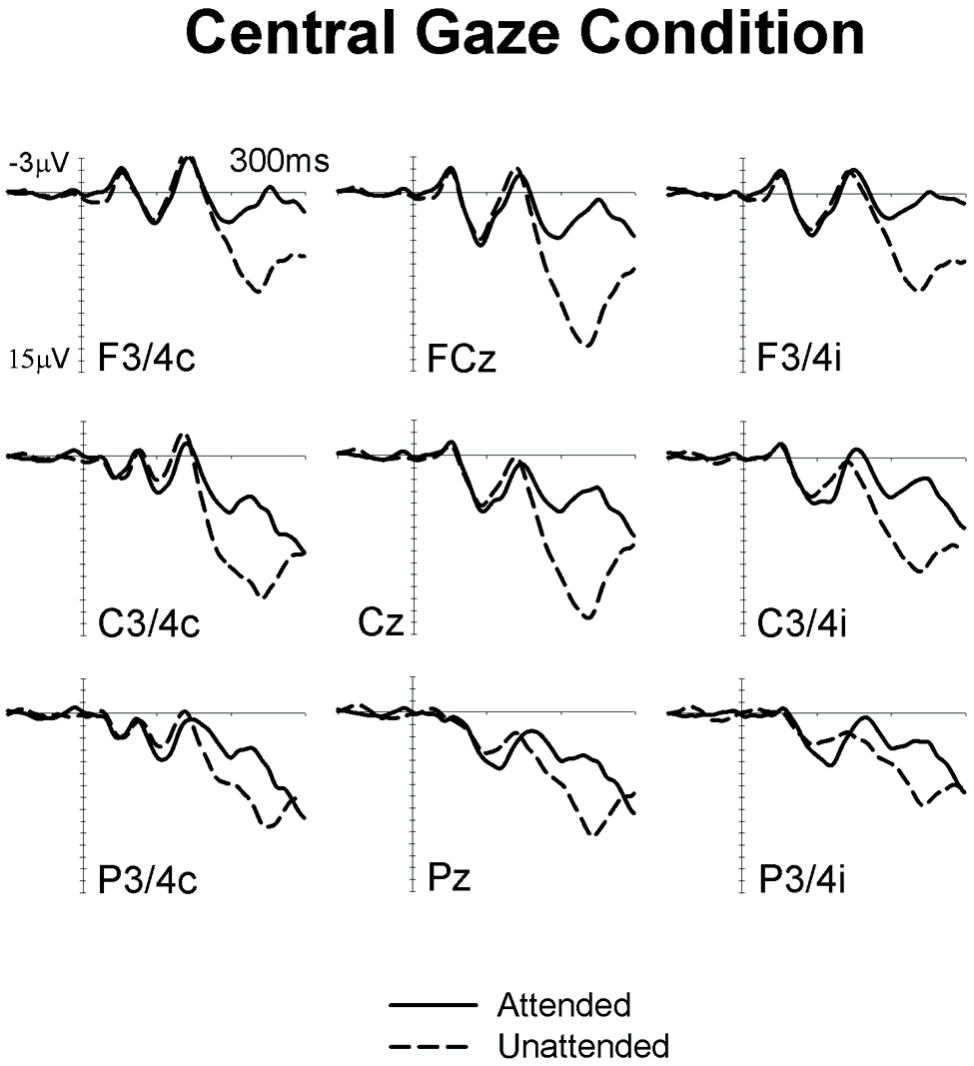
**Grand-averaged somatosensory ERPs elicited in the Central gaze condition at midline electrodes, and at sites contralateral (C) and ipsilateral (I) to the stimulated hand, by tactile non-target stimuli presented to the attended hand (solid lines) and to the unattended hand (dashed lines) in the 300 ms following stimulus onset (relative to a 100 ms pre-stimulus baseline)**.

**FIGURE 3:**
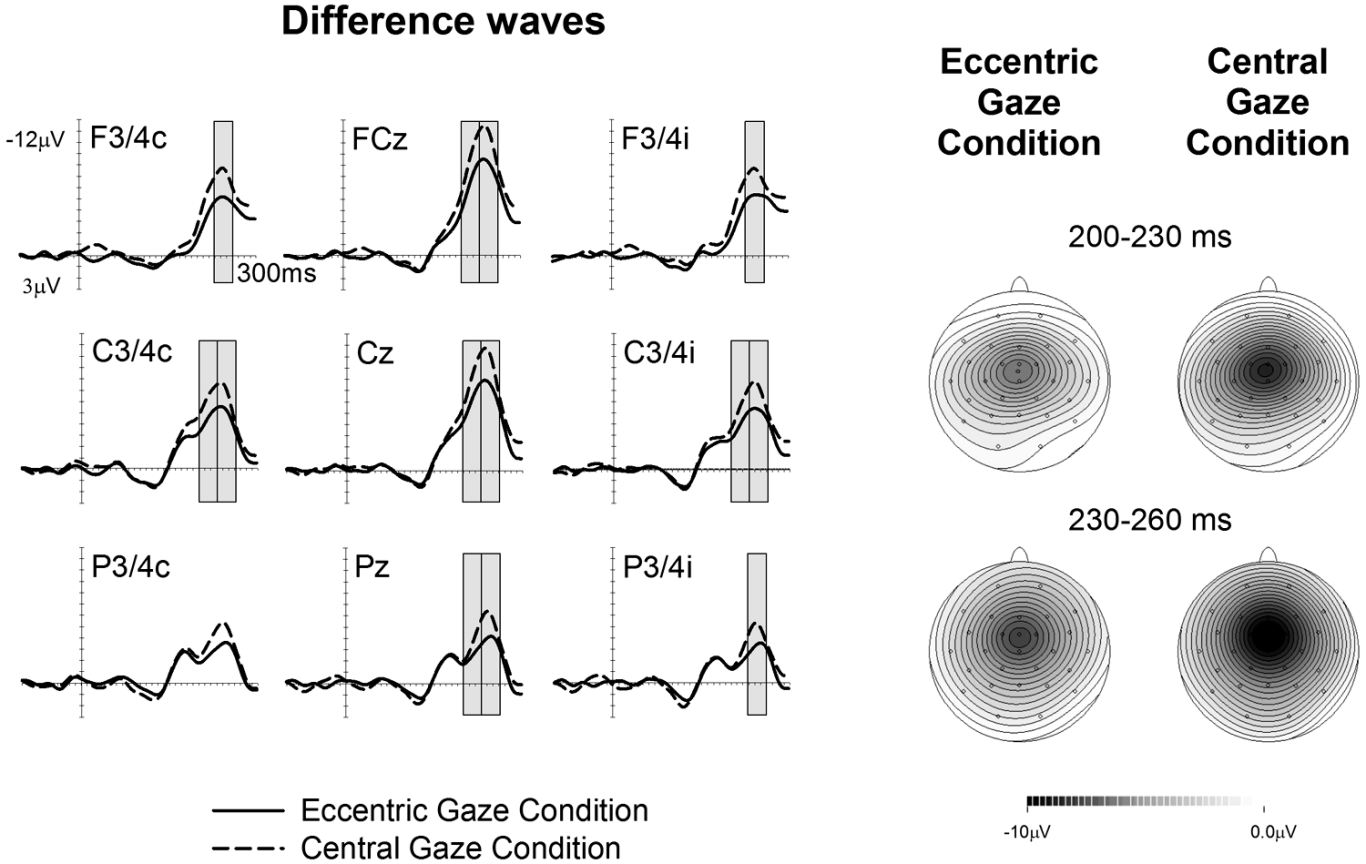
**Difference waveforms are obtained by subtracting the ERPs elicited by tactile non-target stimuli presented to the unattended hand from ERPs elicited by stimuli presented to the attended hand, with solid vs. dashed lines indicating the Eccentric gaze and Central gaze conditions, respectively.** Gray boxes represent the time windows in which significant differences were observed between attentional modulations in the Eccentric and Central gaze conditions. The corresponding topographical voltage maps of attention effects for tactile ERPs are shown separately for the Eccentric gaze and Central gaze conditions for the 200–230 ms, and 230–260 ms intervals after stimulus onset, and are computed by spherical spline interpolation. Amplitude scales range between –10 and 0 μV for the Eccentric gaze condition and –10 to 0 μV for the Central gaze condition.

**Figures 1** and **2** show ERPs elicited in the Eccentric gaze and Central gaze conditions, respectively, by non-target stimuli delivered to the attended (solid line) and unattended (dashed line) hands in the 300 ms interval post-stimulus at F3∖4, C3∖4, P3∖4 recording sites, displayed separately for electrodes ipsilateral (right) and contralateral (left) to the stimulated hand, as well as at midline electrodes Fcz, Cz, and Pz. Attentional modulations are further illustrated in **Figure 3**, where the corresponding attended-minus-unattended difference waveforms are shown (left panels), with solid vs. dashed lines in those panels differentiating the Eccentric and Central gaze conditions, respectively. In both conditions, sustained spatial attention had systematic effects on somatosensory ERPs, with enhanced mid-latency components followed by a sustained attentional negativity for tactile stimuli at attended as compared to unattended locations. Although these effects were present for both tasks, late enhanced negativities appeared considerably larger in the Central gaze than in the Eccentric gaze conditions.

Significant main effects of ***attention*** emerged in all time windows between 80 and 260 ms after stimulus onset. An early positivity for attended relative to unattended stimuli was found at central electrodes ipsilateral and contralateral to the stimulated hand in the 80–110 ms time window [both sites *F*(1,17) > 6.2, *p* < 0.023, $\eta_{p}^{2}$ > 0.27]. Analogous effects were present at all electrodes sites in the following 110–140 ms interval [all sites *F*(1,17) > 6.7, *p* < 0.019, $\eta_{p}^{2}$ > 0.28]. Between 140 and 260 ms, sustained attention was reflected by enhanced negativities in response to tactile stimuli at attended vs. unattended locations. In the 140–170 ms interval, significant main effects of attention were found at contralateral and ipsilateral posterior electrodes [both sites *F*(1,17) > 6.9, *p* < 0.018, $\eta_{p}^{2}$ > 0.29] as well as ipsilateral central electrodes [*F*(1,17) = 7.3, *p* = 0.015, $\eta_{p}^{2}$ = 0.3]. In the following 170–200, 200–230, and 230–260 ms intervals main effects of attention were reliably present at all electrode sites, [all sites *F*(1,17) > 5.3, *p* < 0.035, $\eta_{p}^{2}$ > 0.24; except for ipsilateral anterior sites in the 170–200 ms interval, where this effect did not reach significance, *F*(1,17) = 3, *p* = 0.1, $\eta_{p}^{2}$ = 0.15].

A main effect of ***gaze direction*** emerged between 200–230 and 230–260 ms time interval at central electrodes ipsilateral and contralateral to the stimulated hand as well as midline electrodes [all *F*(1,17) > 5.03, *p* < 0.039, $\eta_{p}^{2}$ > 0.23], revealing that ERPs elicited in the Eccentric gaze condition were overall more negative than those obtained in the Central gaze condition.

Significant interactions between ***gaze direction and attention*** emerged between 200 and 260 ms post-stimulus, reflecting the fact that in these time windows the effects of sustained attention were reduced in the Eccentric gaze relative to the Central gaze condition (see **Figure 3**). In the 200–230 ms time interval, gaze direction × attention interactions were found at contralateral and ipsilateral central electrodes [both sites *F*(1,17) > 4.6, *p* < 0.046, $\eta_{p}^{2}$ > 0.21] as well as at midline electrodes [*F*(1,17) = 5.5, *p* < 0.032, $\eta_{p}^{2}$ = 0.24]. Between 230 and 260 ms post-stimulus, this interaction was significantly present at all electrode sites, [all *F*(1,17) > 4.5, *p* < 0.049, $\eta_{p}^{2}$ > 0.21 (except for contralateral posterior site, where this effect only approached significance, *F*(1,17) = 4.4; *p* = 0.052, $\eta_{p}^{2}$ = 0.2)]. Follow-up analyses conducted separately for each condition in both time windows, revealed that main effects of attention were present at all sites where significant gaze direction × attention interactions were found, not only in the Central gaze condition [all *F*(1,17) > 11.1, *p* < 0.001, $\eta_{p}^{2}$ > 0.4], but also in the Eccentric gaze condition [all *F*(1,17) > 33.6, *p* < 0.002, $\eta_{p}^{2}$ > 0.66].

**FIGURE 4:**
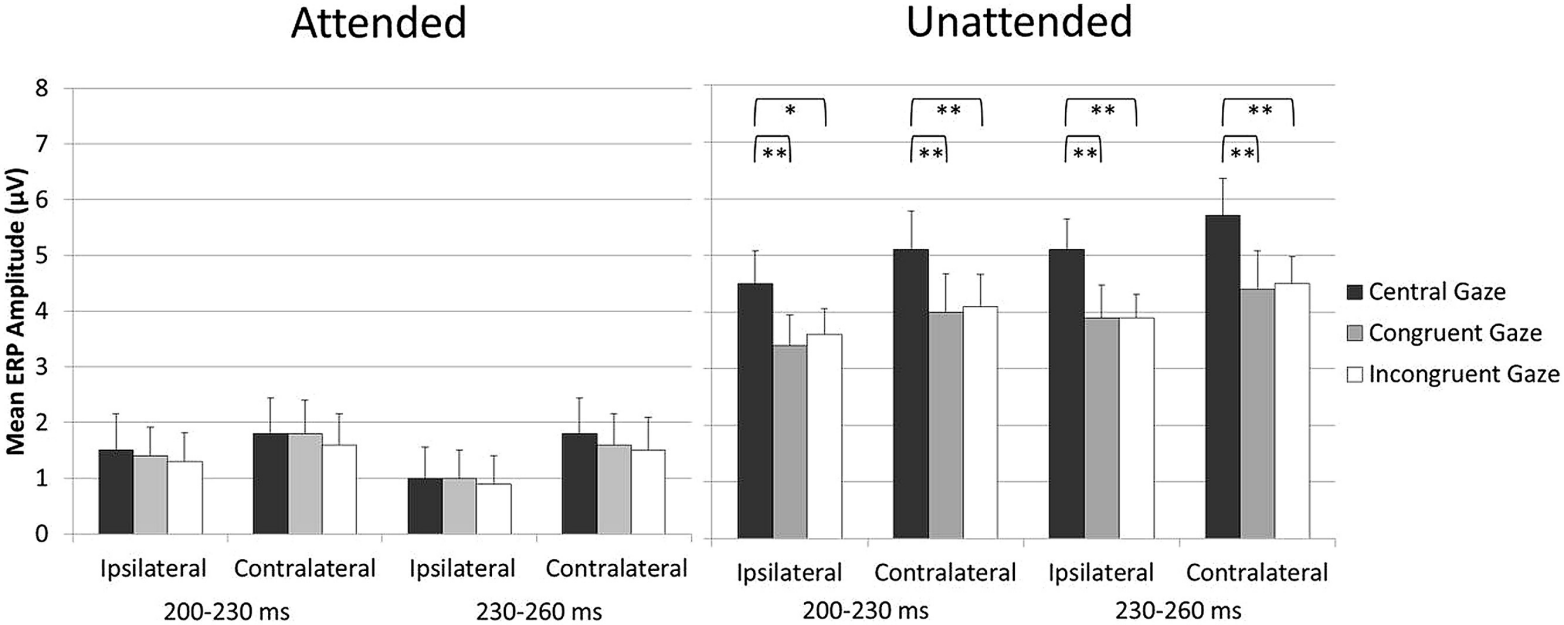
**Mean ERP amplitude elicited by attended stimuli (left panel) and unattended stimuli (right panel) over central electrodes ipsilateral and contralateral to the stimulated hand in the 200- to 260-ms time interval after stimulus onset, shown separately for the Central, Congruent, and Incongruent gaze conditions (error bars represent standard errors).** Significant and highly significant pairwise comparisons are indicated with “∗” and “∗∗,” respectively.

Because the aim of the present study was to assess any impact of holding gaze on an eccentric vs. central fixation on tactile attention, blocks in which participants were gazing laterally toward the side of the task-relevant hand and blocks in which participants were gazing laterally to the side of the task-irrelevant hand were collapsed across. In this way, any direct effect of gaze on tactile processing was canceled out. However, we have recently demonstrated that both gaze and attention can have a direct modulatory effect on tactile processing (Gherri and Forster, 2012b). Therefore, one might ask whether there are any “direct” effects of gaze on touch in the present experiment and whether these might be at least in part responsible for the reduction of the attentional modulations in the Eccentric as compared to the Central gaze conditions observed in the 200–260 ms interval, see **Figure 3**. To address this question, an additional analysis was carried out in which the Eccentric gaze condition was split into two different conditions defined according to the instructed allocation of gaze and tactile attention in the different blocks: the Congruent gaze condition (in which gaze and tactile attention were directed to the same position in space) and the Incongruent gaze condition in which gaze was directed to one side and attention to the opposite side of space). The analysis was identical to the ones reported above except that the factor gaze direction had now three levels rather than two (Central gaze vs. Congruent gaze vs. Incongruent gaze). Mean amplitude values are shown in **Figure 4**. Significant gaze direction × attention interactions were observed between 200 and 260 ms post-stimulus over central electrodes contralateral and ipsilateral to the stimulated hand [all *F*(2,34) = 3.9, *p* < 0.03, $\eta_{p}^{2}$ > 0.19; except for ipsilateral electrodes in the 200–230 ms time window which only approached significance *F*(2,34) = 2.8, *p* = 0.075, $\eta_{p}^{2}$ = 0.14]. Follow-up analyses were conducted separately for ERPs elicited by attended and unattended stimuli (200–230 and 230–260 ms post-stimulus) at ipsilateral and contralateral central electrodes. ERPs elicited by attended stimuli were not significantly affected by gaze direction, **Figure 4** left panel [all *F*(2,34) < 1, *p* > 0.7, $\eta_{p}^{2}$ < 0.02]. In contrast, ERPs elicited by unattended stimuli systematically differed as a function of gaze direction, **Figure 4** right panel [all *F*(2,34) > 6.6, *p* < 0.005, $\eta_{p}^{2}$ > 0.28]. Further analyses revealed that mean ERP amplitudes elicited by unattended stimuli were larger in the Central gaze condition as compared to both Congruent and Incongruent gaze conditions [all *F*(1,17) > 6, *p* < 0.025, $\eta_{p}^{2}$ > 0.26], while no significant difference emerged between ERPs measured in the Congruent and Incongruent gaze conditions [all *F*(1,17) < 1.1, *p* > 0.3, $\eta_{p}^{2}$ < 0.06].

Overall, the results of this additional analysis revealed that similar ERPs were elicited by “gazed” and “non-gazed” attended stimuli (e.g., attended stimuli in the Congruent and Incongruent gaze conditions, respectively), as well as by “gazed” and “non-gazed” unattended stimuli (e.g., unattended stimuli in the Incongruent and Congruent gaze conditions, respectively) in the 200–260 ms interval^3^. Importantly for the aim of this study, this indicates that similar attentional modulations were observed when gaze was directed laterally to the side, regardless of whether it was directed toward the side of the task-relevant or the task-irrelevant hand. It is therefore possible to rule out the hypothesis that direct effects of gaze on touch were in part responsible for the results reported in the main analysis, namely the reduction of the attentional modulations in the Eccentric as compared to the Central gaze conditions observed in the 200–260 ms interval.

## GENERAL DISCUSSION

External spatial representations encode the current location of the body in external space. This is achieved trough the integration of visual information about the body and its environment with proprioceptive and vestibular information about the location of the different body parts. Previous studies demonstrated that visual information plays a crucial role in the development of these external representations (c.f. Röder et al., 2004, 2008; but see also Eardley and van Velzen, 2011), and suggested that eye position might serve as a reference point to encode relevant body locations in external space (Schicke and Röder, 2006). If the external representations that mediate tactile attention are based at least in part on the position of the eye in the orbit, attentional modulations of tactile processing should be affected by manipulations of gaze direction.

To test this hypothesis, we instructed participants to focus their tactile attention on the task-relevant hand in order to respond to infrequent targets (gap stimuli) presented to that hand (sustained attention task), while ignoring target stimuli presented to the task-irrelevant hand. In addition, tactile non-target stimuli (no gap stimuli) presented to either hand was to be ignored. Because the tactile target/non-target discrimination was a difficult one (target and non-target stimuli were physically identical for the first 97 ms of presentation, after which only target stimuli had a 6 ms gap), participants were strongly encouraged to focus their tactile attention on the task-relevant hand, where they had to respond to infrequent targets. Eye-in-the-orbit position was manipulated by asking participants to gaze either laterally to an eccentric fixation point (Eccentric gaze condition) or to a central fixation (Central gaze condition) while performing the same sustained attention task. ERPs elicited by tactile non-target stimuli presented to the attended and unattended hand were compared as a function of gaze direction (Eccentric vs. Central gaze).

In line with previous ERP studies on tactile spatial attention (e.g., Desmedt and Robertson, 1977; Michie, 1984; Michie et al., 1987; García-Larrea et al., 1995), we observed systematic modulations of somatosensory ERPs elicited by non-target stimuli delivered to the currently attended and unattended hand. More specifically, tactile attention resulted in enhanced positivities for tactile stimuli presented to the attended hand in the P100 time-range. These effects were followed by enhanced negativities for stimuli at attended locations, overlapping with the descending flank of the N140 component and the subsequent sustained processing negativity (Nd). Importantly, these effects were reliably present not only in the Central gaze condition (which is directly comparable with tactile attention tasks used in previous studies), but also in the Eccentric gaze condition. This observation demonstrates the presence of similar operations of tactile spatial attention in both conditions.

However, and crucially for the aim of this study, we also observed systematic differences between the attentional modulations in the Eccentric and Central gaze conditions. More specifically, between 200 and 260 ms post-stimulus the magnitude of the tactile attentional modulation was reduced when the eyes were directed laterally to the side (Eccentric gaze) as compared to when gaze was directed to the center (Central gaze). This difference between gaze conditions was observed in the time range of the Nd wave related to tactile spatial attention, which reflects processing in and beyond somatosensory cortex and has been suggested to reflect the in-depth processing of task-relevant features of attended stimuli (cf. Michie, 1984).

The fact that attentional modulations of somatosensory ERPs were systematically influenced by gaze direction demonstrates for the first time that tactile spatial attention is sensitive to the position of the eye-in-orbit. This suggests that the direction of gaze might serve as a reference point against which relevant body locations are encoded for external representations of space. Importantly, the observation that the attentional modulations of tactile processing are reduced in the Eccentric gaze condition indicates that tactile selectivity is hindered by an eccentric position of the eye in the orbit. Thus, tactile selectivity appears to be less efficient when gaze is directed away from the central fixation point.

Previous tactile attention studies suggested that when there is a conflict between different spatial representations defining a task-relevant location, the operation of spatial attention is disrupted. This is typically observed in attention tasks when participants cross their hands. Responses to tactile stimuli presented to the attended hand are slower and attentional modulations of somatosensory ERP components in response to tactile stimuli are delayed and attenuated with crossed as compared to uncrossed hands (Eimer et al., 2001, 2003; Röder et al., 2008; Eardley and van Velzen, 2011; Gherri and Forster, 2012a). More specifically, in the crossed hands condition, the attentional modulations of the mid-latency components were absent and the subsequent sustained attentional negativity, Nd, was reduced in amplitude relative to the uncrossed hands condition. This impairment with crossed hands has been interpreted as indicating the presence of two opposite and competing spatial representations used by the brain to encode the location of the task-relevant hand, the somatotopic and the external representations. Thus, attentional modulations of somatosensory processes are attenuated under conditions in which body posture manipulation results in conflicts between different spatial representations (but see also Heed and Röder, 2010, for a different explanation of the mechanisms underlying the hands crossing effects on tactile spatial attention).

In the present study, the hands were the task-relevant body part but their location was held constant throughout the experiment. Instead, the eye position was manipulated (Eccentric vs. Central gaze). In the Central gaze condition, the eyes were aligned with the head, trunk, and body midline as well as with the central fixation point. In contrast, in the Eccentric gaze condition the eyes were directed laterally and diverted from the central positions of head, trunk, and body midline. Thus, in the Eccentric gaze condition the eye-centered external representation was no longer in spatial register with the somatotopic representation. For instance, when participants’ gaze was directed toward the left fixation point, the origin of the external eye-centered representation shifted accordingly toward the left side of space. Under these circumstances, the left hand (located on the left side close to the left fixation) is coded as “center” according to an external eye-centered representation, whereas it is coded as “left” according to a somatotopic representation. The results of the present study suggest that directing the eyes toward an eccentric position is sufficient to create a misalignment between external and somatotopic frames of references which interferes with the operations of tactile attention.

One interesting aspect of our results concerns the time course of the effects of body posture on tactile selectivity. The manipulation of the eye-in-orbit position affected the attentional modulations of touch at relatively late stages of processing (beyond 200 ms post-stimulus). In contrast, the manipulation of the hands location in external space (as in previous crossed vs. uncrossed hands studies) resulted in modulations of the effects of spatial attention on tactile processing already within modality-specific somatosensory brain areas (Eimer et al., 2001, 2003; Röder et al., 2008; Eardley and van Velzen, 2011; Gherri and Forster, 2012a). Thus, it appears that the conflict between somatotopic and external codes (uncrossed vs. crossed hands manipulation) affects tactile selectivity earlier than the misalignment between these representations (eccentric vs. central gaze direction). This pattern of results might suggest that the strength of the spatial incongruence between different representations is reflected by the onset time of the modulations of the effects of spatial attention on tactile processing under different body posture conditions (with earlier disruption for stronger spatial incongruence). While this is an intriguing possibility, it is to note that the specific attention task used in these studies (sustained vs. transient attention tasks) might also be in part responsible for these differences (Eimer and Forster, 2003). In the present study, a sustained attention task was used in which participants had to maintain their attention on the task-relevant hand throughout a block of trials. In contrast, in previous uncrossed vs. crossed hands studies participants performed spatial cuing tasks in which the task-relevant hand was cued on a trial by trial basis. It is therefore possible that an earlier attentional disruption was observed in the transient attention tasks because the spatial incongruence between somatotopic and external representations had to be solved on each trial before the task-relevant hand could be selected, whereas the spatial incongruence due to the eye-in-orbit manipulation was in part solved or adjusted for during the block of trials in which tactile attention was held on the same task-relevant hand. Future studies should directly investigate whether body posture manipulations involving the hands and the eye positions still result in different modulations of the attention effects on touch under conditions in which participants perform the same attention task.

Overall, our study has provided the first indication that eye position might be used as a reference point to encode the position of other body parts in external space during tactile attention tasks. However, it is worth noting that eye position might not be the only reference point for external representations that mediate tactile spatial attention. For instance, the role of eye and head direction has been systematically investigated in a series of studies on tactile localization demonstrating that relevant body locations can be coded in external space according to several different reference points (Ho and Spence, 2007; Harrar and Harris, 2009; Pritchett and Harris, 2011; Pritchett et al., 2012; Mueller and Fiehler, 2014). When participants were asked to localize the position of a tactile stimulus presented to the participants’ body, the presence of systematic eye-position and head-position related errors revealed that not only the eye but also the head position contributed to the representation of external space (Ho and Spence, 2007; Harrar and Harris, 2009; Pritchett and Harris, 2011; Pritchett et al., 2012). In addition, also the body midline might be used as a reference point to code tactile locations under specific circumstances (Pritchett et al., 2012). These results suggest the existence of multiple reference points for external representations of body locations. While it is not known whether analogous spatial reference frames are activated during localization tasks and the endogenous selection of relevant body locations in touch, it is conceivable to suppose that tactile attention might operate on multiple external representations of the body, each one anchored to a different reference point. If multiple external representations mediate tactile selectivity, it is likely that in the Eccentric gaze condition of the present experiment the external eye-centered representation was not only spatially incongruent with the somatotopic representation but also with other external representations of body locations such as head- or trunk-centered representations. Future studies should directly investigate whether tactile spatial attention is mediated by multiple external representations by systematically manipulating the position of the task-relevant body parts with respect to the different external reference points and whether the effects of the spatial incongruence between external and somatotopic representations on tactile attention are different from those of the incongruence between different external representations.

In summary, our results confirm that the attentional selection of one hand vs. the other is strongly affected by changes in body posture and expand previous research on body posture manipulations indicating that also the position of the eye in the orbit can modulate tactile spatial attention. These findings demonstrate for the first time that the efficiency of spatial attention in touch is at least in part determined by gaze direction. Attentional modulations of somatosensory processes are attenuated under conditions in which gaze is directed toward an eccentric location. This might suggest that an eccentric position of the eyes might be sufficient to create a misalignment between multiple spatial representations of the body that ultimately hinders tactile selectivity.

## Conflict of Interest Statement

The authors declare that the research was conducted in the absence of any commercial or financial relationships that could be construed as a potential conflict of interest.

## Footnotes

1 While some authors consider the terms external and allocentric spatial representations equivalent, we use the term “external representation” to refer to body representations that are independent of body surface (non-somatotopic). Within this broad definition external representations can be egocentric or allocentric, depending on the relevant reference point (e.g., Soto-Faraco et al., 2004).

2 Note that the term gaze typically refers to the combination or sum of the eye and head positions. Here, however, because the head position is maintained centrally and aligned with the body midline throughout the experiment, we will use the term gaze to refer to the eye position only.

3 Pairwise comparisons between ERPs elicited by attended stimuli in the Congruent and Incongruent gaze conditions conducted separately for the 200–230 and 230–260 ms time windows were found to be statistically non-significant at ipsilateral [both *t*(17) < 0.3, *p* > 0.7, *d* < 0.08] and contralateral [both *t*(17) < 0.7, *p* > 0.45, *d* < 0.18] central electrodes. In a similar way, comparisons between unattended stimuli ERPs elicited in the Congruent and Incongruent gaze conditions were not significant at central electrodes ipsilateral [both *t*(17) < 1, *p* > 0.3, *d* < 0.24] and contralateral [both *t*(17) < 0.5, *p* > 0.6, *d* < 0.12] to the stimulated hand. While these results suggest that similar ERPs were elicited by “gazed” and “non-gazed” tactile stimuli for both attended and unattended conditions in this specific time window, these are null results and should be interpreted with caution. The effect sizes of these effects of gaze (Coehn’s *d*) varied between 0.001 and 0.24. A *post hoc* power analysis (80% chance, significant at the 5% level) was carried out using the Gpower program (Faul et al., 2007) and revealed that a sample of 139 participants would be required to detect a small effect size (*d* = 0.24), while a sample of 7,848,863 participants would be necessary to detect a very small effect (such as *d* = 0.001). Based on this analysis, we cannot therefore rule out the possibility that an effect of gaze would not have been detected with a substantially larger sample. However, it is relevant to point out that a direct effect of gaze on tactile processing was observed in Gherri and Forster (2012b) with an analogous sample (*N* = 18) but in an earlier time window (mid-latency somatosensory ERP components) and this effect was no longer present beyond 200 ms post-stimulus. We therefore suggest that the small effect sizes reported above simply reflect the fact that the direct effect of gaze on touch is not reliably present beyond 200 ms post-stimulus.
